# Effect of bolus tracking region-of-interest position within the descending aorta on luminal enhancement of coronary arteries in coronary computed tomography angiography

**DOI:** 10.1097/MD.0000000000015538

**Published:** 2019-05-13

**Authors:** Ryo Kurokawa, Eriko Maeda, Harushi Mori, Shiori Amemiya, Jiro Sato, Kenji Ino, Rumiko Torigoe, Osamu Abe

**Affiliations:** a Department of Radiology, Graduate School of Medicine, University of Tokyo; b Department of Radiation Technology, University of Tokyo Hospital; c Canon Medical Systems Corporation, Tokyo, Japan.

**Keywords:** bolus tracking technique, coronary artery, coronary computed tomography angiography

## Abstract

To compare coronary artery luminal enhancement in coronary computed tomography angiography (CCTA) between ventral and dorsal region-of-interest (ROI) bolus tracking in the descending aorta.

The records of 165 consecutive patients who underwent CCTA with non-helical acquisition from July 2017 to March 2018 were retrospectively examined. We performed 320-row CCTA with bolus tracking [scan triggered at 260 HU in the descending aorta] and 133 patients were finally included. ROI was set in the ventral and dorsal halves of the descending aorta in 68 and 65 patients, respectively.

Contrast arrival time was significantly shorter in the dorsal group (ventral: 21.8 ± 0.372 s; dorsal: 20.7 ± 0.369; *P* = .0295). The mean density of the proximal and distal RCA was significantly higher in the ventral group (proximal: ventral, 428.1 ± 6.95 HU; dorsal, 405.5 ± 7.72 HU, *P* = .0318; distal: ventral, 418.0 ± 9.29 HU; dorsal, 393.2 ± 9.46 HU, *P* = .0133).

Dorsal bolus tracking ROI in the descending thoracic aorta significantly reduced preparation time and RCA CT values.

## 1 Introduction

Coronary computed tomography angiography (CCTA) is a noninvasive technique to detect coronary artery disease, which is characterized by its high sensitivity and negative predictive values.^[1,2]^ Significant correlations between CCTA and invasive coronary angiography for the results of coronary luminal stenosis and fractional flow reserve have been demonstrated.^[3,4]^ Recently, CCTA was shown to reduce the future risk of myocardial infarction.^[5]^ Bolus-tracking methods with regions-of-interest (ROIs) in the aorta have been adopted to determine scan timing in CCTA. It is necessary to move ROIs manually in line with body movement from patient respiration; therefore, it is common for bolus-tracking ROIs to be placed in the ascending or descending aorta to avoid displacement of the ROIs by breath movement.^[6]^ In CCTA, motion artifact,^[7]^ coronary calcification,^[8]^ and coronary arterial enhancement^[9]^ are factors affecting image quality. Among these, coronary enhancement is strongly affected by dose, concentration, injection rate, injection duration, and scan delay.^[10–12]^ Imaging protocols can determine these factors and take them into account; however, for bolus-tracking methods in which an increase in CT value in the ROI is the trigger for the start of imaging, there has been little discussion on the position of ROIs in the aorta.

**Figure 1:**
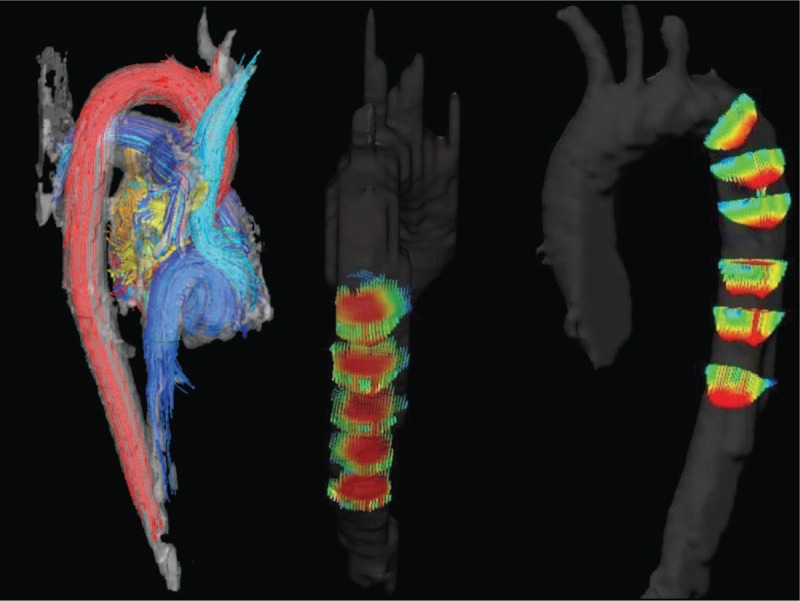
4D-MRI velocity mapping indicates that faster flow (red) is more inclined externally and proximal to the middle of the thoracic descending aorta (images courtesy of K. Itatani). 4D-MRI = 4-dimensional magnetic resonance imaging.

Recent 4-dimensional magnetic resonance imaging (4D-MRI) studies using the phase contrast method have revealed that arterial flow velocity is not uniform along the same axial plane in the aorta.^[13–17]^ This flow varies depending on the location along the axial plane and according to cardiac cycle.^[18–20]^ Using 4D-MRI, Miyazaki et al. have shown a tendency for a faster flow velocity at the dorsal side of the middle thoracic descending aorta (Fig. 1).^[15]^ Therefore, we hypothesized that a dorsally placed ROI would shorten contrast arrival time and potentially cause decreased coronary artery enhancement. In this study, we examined the influence of ROI position in the CCTA of the thoracic descending aorta on scan timing and CT values in the coronary arteries.

## 2 Methods

### 2.1 Ethical approval

This retrospective study was approved by the local ethics committee, and the requirement for informed consent for study participation was waived.

### 2.2 Patients

The records of 165 consecutive patients who underwent CCTA with non-helical acquisition from July 2017 to March 2018 were retrospectively examined. Exclusion criteria were as follows: weight, <42 kg (n = 9); weight, >85 kg (n = 5); scan started manually because of insufficient increase in the ROI enhancement (n = 8); irregular protocol due to congenital anomaly (n = 2); post coronary artery bypass graft (n = 2); ROI placed on aortic arch for a descending thoracic aorta aneurysm (n = 2); aortic dissection (n = 1); dilated cardiomyopathy (n = 1); administration of additional contrast material (n = 1); and cardiac tamponade (n = 1). Finally, 133 patients were included in the analysis. An expert CT technician performed all CT exams, including ROI positioning. Bolus-tracking ROIs were placed manually by the CT technician along the ventral (n = 68) or dorsal (n = 65) halves of the aorta. The ROI was tracked along with chest movement (due to breathing) to maintain the same position within the descending aorta. The ROI position was switched monthly.

Patient characteristics revealed no significant differences between groups (Table 1).

**Table 1:**
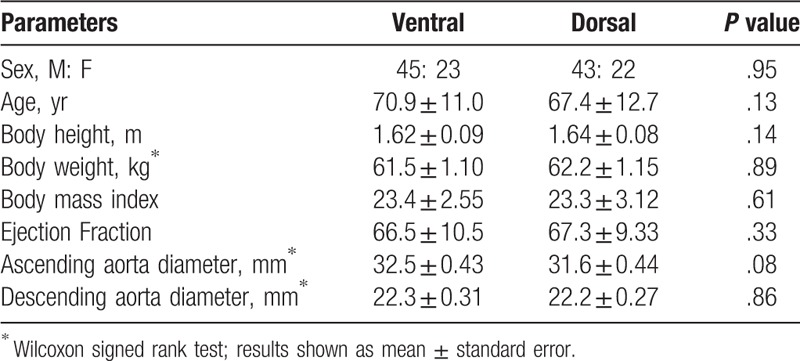
Patient background (mean ± standard deviation).

### 2.3 CT data acquisition

All patients underwent CCTA using a second-generation 320-row CT (Aquilion ONE ViSION edition; Canon, Tochigi, Japan). Prospective electrocardiogram-triggered axial scanning was performed in all patients. CT data acquisition parameters were as follows: detector configuration, 320 × 0.5 mm; tube potential, 120 kVp. An experienced cardiovascular radiologist determined the acquisition window. Tube current was determined according to body weight.

We administered 22.2 mg/kg/s iopamidol (Iopamiron 370, 370 mg/mL; Bayer, Osaka, Japan) to patients. A 10-s contrast medium injection was followed by a 4-s injection of 50:50 mixed contrast media and 30-mL saline flush with the same injection speed as the contrast media. The bolus-tracking method was used using the following protocol: when the left ventricle reached 100 Hounsfield units (HU), patients were instructed to breathe in and hold; when the descending aorta reached 260 HU, scanning was started. The effective radiation dose was derived by multiplying the dose length product by the chest conversion coefficient (k = 0.014 mSv/mGy/cm).

When the heart rate reached 65 beats per minute, an oral β-blocker (20–40 mg metoprolol) was administered in the outpatient department. Two hours later, CCTA was performed. If the heart rate on site exceeded 65 beats per minute, up to 12.5 mg Landiolol (Corebeta; Ono Pharmaceutical, Tokyo, Japan) was administered intravenously. All patients received 2.5 mg sublingual isosorbide dinitrate (Nitorol; Eisai, Tokyo, Japan) before imaging.

For reconstruction, the forward projected model-based Iterative Reconstruction SoluTion (FIRST) cardiac strong algorithm was used. Slice thickness and increment were 0.5 mm and 0.25 mm, respectively.

### 2.4 Image analysis

**Figure 2:**
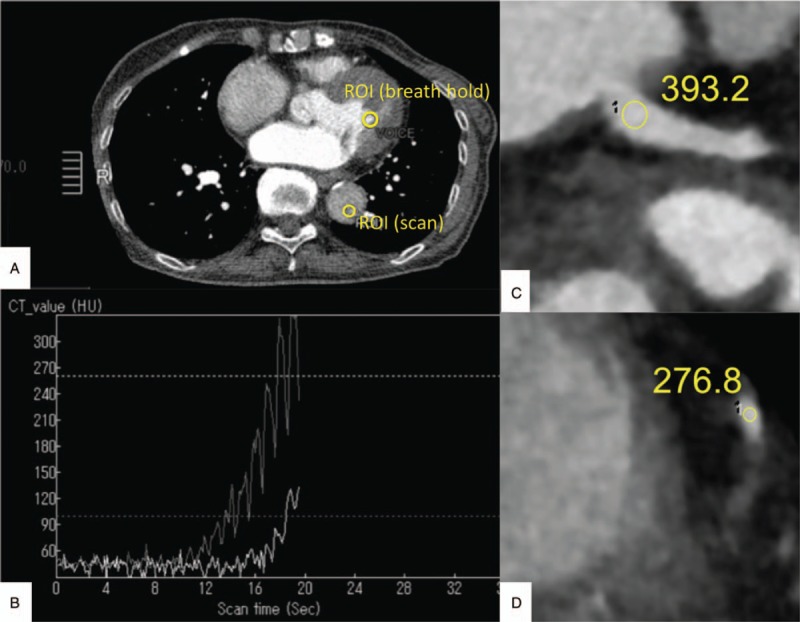
ROI placement and measurement of the coronary artery and preparation time. When the left ventricle reached 100 HU, patients were instructed to breathe in and hold; when the descending aorta reached 260 HU while manually adjusting the ROI position in the dorsal or ventral half of aorta, scanning was started (A, B). A circular ROI was placed in the proximal (C) and distal (D) RCA. HU = Hounsfield units, RCA = right coronary arteries, ROI = region of interest.

A circular ROI was placed manually, carefully avoiding vessel walls, calcifications, or stents, at 2 different sites of the left anterior descending coronary artery (LAD: proximal, left main trunk; distal, middle of #8) and the right coronary arteries (RCA: proximal, RCA base: distal, middle of #3). The CT value gradient (HU/10 mm) was automatically measured using a Vitrea Workstation (Canon, Tochigi, Japan). Coronary arteries with stents and/or calcifications >1 cm and/or stenosis >75% were excluded from the gradient measurement to avoid measurement error. We recorded the contrast arrival time displayed on the CT machine (Fig. 2).

The CT value of the ventral and the dorsal halves of the descending thoracic aorta in plain CT was recorded in order to analyze the effect of the beam-hardening vertebrae artifact.

### 2.5 Statistical analysis

We hypothesized that both groups would have similar contrast arrival times and coronary artery CT values. From this hypothesis, we calculated that the minimum sample to detect a difference of 15 HU, or 1 s, was 128 arteries (approximately 22 patients) or 128 patients at 0.8 power. Therefore, we included 133 patients (ventral: n = 68; dorsal: n = 65) in this study.

Results were considered statistically significant if *P* was <.05. Statistical analyses were performed using JMP software (version 13.2.0; SAS, Cary, NC). Contrast arrival time, coronary artery CT values, and CT value gradients were compared using Mann–Whitney *U* tests.

## 3 Results

**Figure 3:**
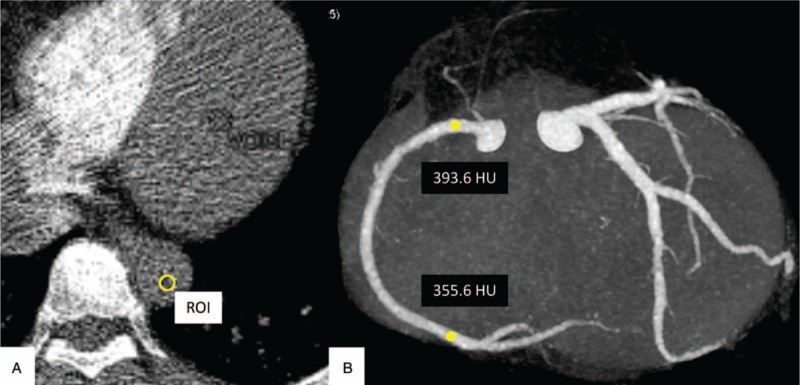
Example case of a 46-year-old woman with a dorsally placed ROI. CT values of the proximal and the distal RCA were 393.6 HU and 355.6 HU, respectively. Contrast arrival time was 18.65 seconds. CT = computed tomography, HU = Hounsfield units, RCA = right coronary arteries, ROI = region of interest.

**Figure 4:**
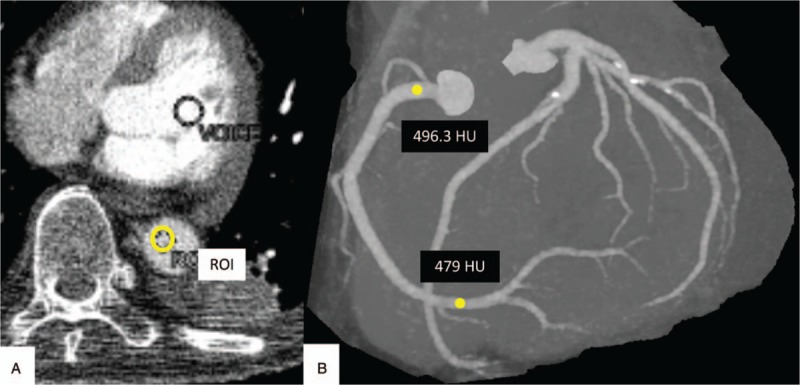
Example case of a 66-year-old man with a ventrally placed ROI. CT values of the proximal and the distal RCA were 496.3 HU and 479.0 HU, respectively. Contrast arrival time was 26.84 seconds. CT = computed tomography, HU = Hounsfield units, RCA = right coronary arteries, ROI = region of interest.

Contrast arrival time was significantly shorter in the dorsal group than the ventral group (ventral: 21.8 ± 0.372 s; dorsal: 20.7 ± 0.369; *P* = .0295). The proximal LAD (LMT) CT value showed a tendency to be lower in the dorsal group (proximal: ventral, 435.2 ± 7.37 HU; dorsal, 415.6 ± 7.52 HU, *P* = .0653; distal: ventral, 383.1 ± 8.45 HU; dorsal, 365.7 ± 9.61 HU, *P* = .222); however, the RCA CT value was significantly lower in the dorsal group in the proximal and distal regions (proximal: ventral, 428.1 ± 6.95 HU; dorsal, 405.5 ± 7.72 HU, *P* = .0318; distal: ventral, 418.0 ± 9.29 HU; dorsal, 393.2 ± 9.46 HU, *P* = .0133) (Fig. 3 and Fig. 4).

There were no significant differences in CT value gradients between groups (ventral: 37.6 HU ± 15.7; dorsal: 35.6 HU ± 12.8, *P* = .2532). These results are summarized in Table 2. The radiation dose results are summarized in Table 3.

**Table 2:**
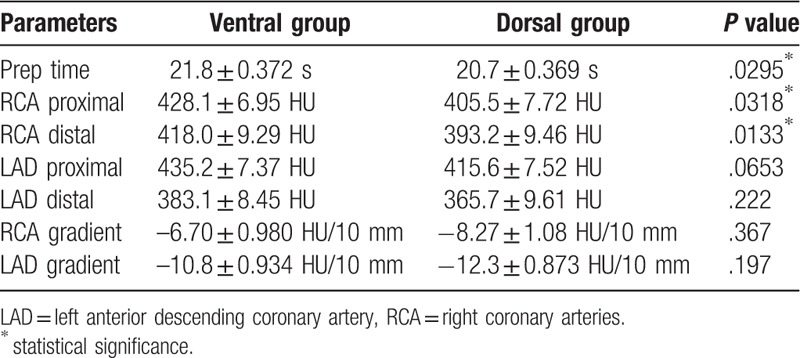
Subjective image analysis (mean ± standard error).

**Table 3:**
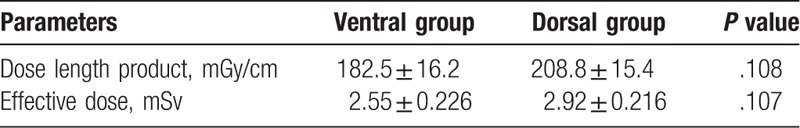
Radiation doses (mean ± standard error).

## 4 Discussion

This is the first study to compare contrast arrival time and luminal enhancement of the coronary arteries in CCTA between dorsal and ventral ROIs of the bolus-tracking ROI in the descending thoracic aorta.

We found that contrast arrival time was significantly shorter when the ROI was placed in the dorsal half of the descending thoracic aorta. Furthermore, this reduced the RCA CT values. LAD CT values also showed a tendency for lower values in the dorsal group. The shorter contrast arrival time caused the lower coronary CT value in the dorsal group. The CT value gradient did not differ between groups, while luminal enhancements of the coronary arteries in the ventral group were significantly higher. This indicated that ROIs should be placed ventrally to ensure optimal coronary enhancement. The ventrally placed ROI also reduces the possibility that the ROIs protrude from the aorta due to body movement from respiration.

Weininger et al suggested in their review on CCTA, that 3 different factors should be considered for the optimization of contrast medium delivery and image acquisition strategies; CT-related factors, patient-related factors, and contrast-related factors.^[12]^ CT-related factors include scan delay, scan direction, and radiation dose. Patient-related factors include body size and cardiac output. Contrast-related factors include injection rate, velocity, protocol, and bolus shape. Moradi et al also reviewed multiple factors affecting CCTA imaging quality and emphasized that cardiac function is the most important factor affecting the timing of contrast material enhancement.^[13]^ However, the importance of the placement of ROIs has been underestimated.

CT values in the aorta on the same plane are not uniform. They vary depending on the region and the cardiac cycle.^[14–21]^ It has been suggested that aortic flow external to the arch tends to be faster than flow internal to proximal region of the thoracic descending aorta, due to centrifugal force.^[15,20,22]^ In addition, retrograde flow is found inside the aortic arch in the early diastolic phase.^[14,16,20]^ This leads to the hypothesis that the influence of stronger retrograde flow on the ventral side is still stronger in the descending thoracic aorta; therefore, the arrival of the contrast agent on the ventral side may become more delayed. Based on these findings, we hypothesized that changing the position of the ROI could affect coronary arrival time and coronary artery enhancement.

Other possible mechanisms that may explain the shorter coronary arrival time in the dorsal group are as follows: first, the effect of beam-hardening artifacts from the vertebrae; second, the specific gravity of the contrast medium-induced dorsal distribution of the hyperdense flow.

We considered that beam-hardening artifacts from the vertebrae could increase the CT values of the dorsal aorta, then attributed to an earlier threshold CT value. However, *t* tests revealed no difference in the intra-aortic CT value in the plain CT between the ventral and dorsal descending thoracic aorta. Based on these results, the first hypothesis did not hold.

The contrast agent we used had a higher molecular weight than blood; therefore, it is more likely to settle dorsally due to gravity and it may reach the threshold of the dorsal CT value earlier. Performing CCTA in the prone or lateral decubitus position may lead to different contrast agent kinetics, but in the present study, all CCTA examinations were performed in the supine position. Further studies are required to confirm the gravitational effect on distribution of the contrast medium.

There are several limitations to this study. First, the level of the patients’ cardiac function was not uniform. The aortic flow rate and speed of the contrast agent are strongly influenced by cardiac function; however, there was no significant difference in cardiac function between groups, which indicates that the difference in flow velocity and contrast medium arrival time due to the cardiac function can be ignored. Second, we performed CCTA while manually adjusting the position of the ROI in the aorta; therefore, there may be some error in dimensional accuracy. However, we minimized this risk by assigning all CT examinations to an experienced CT technician.

In conclusion, bolus-tracking ROIs in the CCTA in the dorsal descending thoracic aorta reduced contrast arrival time and led to lower CT values in the coronary artery. To achieve better coronary artery enhancement, bolus-tracking ROI should be placed ventrally.

## Acknowledgments

We thank Dr. Keiichi Itatani for providing the images for Fig. 1.

## Author contributions

**Conceptualization:** Eriko Maeda, Shiori Amemiya, Jiro Sato.

**Data curation:** Ryo Kurokawa, Kenji Ino.

**Formal analysis:** Ryo Kurokawa.

**Resources:** Rumiko Torigoe.

**Software:** Kenji Ino, Rumiko Torigoe.

**Supervision:** Eriko Maeda, Harushi Mori, Jiro Sato, Osamu Abe.

**Writing – original draft:** Ryo Kurokawa.

**Writing – review & editing:** Ryo Kurokawa, Eriko Maeda, Harushi Mori, Shiori Amemiya, Osamu Abe.

Ryo Kurokawa orcid: 0000-0002-1186-8900.
